# Effect of Temperature on the Expression of Classical Enterotoxin Genes among Staphylococci Associated with Bovine Mastitis

**DOI:** 10.3390/pathogens10080975

**Published:** 2021-08-02

**Authors:** Theeyathart Homsombat, Sukolrat Boonyayatra, Nattakarn Awaiwanont, Duangporn Pichpol

**Affiliations:** 1 Master’s Degree Program in Veterinary Science, Department of Food Animal Clinic, Faculty of Veterinary Medicine, Chiang Mai University, Chiang Mai 50100, Thailand; theeyathart@gmail.com; 2 Department of Food Animal Clinic, Faculty of Veterinary Medicine, Chiang Mai University, Chiang Mai 50100, Thailand; nattakarn.a@cmu.ac.th; 3 Research Group for Veterinary Public Health, Faculty of Veterinary Medicine, Chiang Mai University, Chiang Mai 50100, Thailand; duangporn.p@cmu.ac.th; 4 Department of Veterinary Biosciences and Veterinary Public Health, Faculty of Veterinary Medicine, Chiang Mai University, Chiang Mai 50100, Thailand

**Keywords:** classical enterotoxin gene, gene expression, *Staphylococcus aureus*, coagulase-negative staphylococci, bovine mastitis, staphylococcal food poisoning

## Abstract

Staphylococcal food poisoning (SFP), caused by the contamination of staphylococcal enterotoxins, is a common foodborne disease worldwide. The aims of this study were: (1) to investigate classical staphylococcal enterotoxin genes, *sea, seb*, *sec, sed*, and *see*, among *Staphylococcus aureus* and coagulase-negative staphylococci (CNS) associated with bovine mastitis; (2) to determine the effect of temperature on the expression of classical staphylococcal enterotoxin genes in staphylococci in milk. The detection of classical staphylococcal enterotoxin genes was performed using *S. aureus* (*n* = 51) and CNS (*n* = 47). The expression of classical enterotoxin genes, including *sea, seb, sec,* and *see*, was determined during the growth of staphylococci in milk subjected to ultra-high-temperature processing at two different temperatures: 8 °C and room temperature. Classical staphylococcal enterotoxin genes were expressed more frequently in *S. aureus* (35.30%) than in CNS (12.77%). The *sec* gene was most frequently detected in *S. aureus* (29.41%) and CNS (6.38%). Moreover, the expression of *sea* and *sec* was significantly higher at room temperature than at 8 °C after 16 h of incubation (*p* < 0.05). These results emphasize the importance of maintaining the storage temperature of milk below 8 °C to reduce the risk of SFP.

## 1. Introduction

Staphylococcal food poisoning (SFP) is one of the most common foodborne diseases worldwide. It is caused by the consumption of food contaminated with staphylococcal enterotoxins (SEs) secreted by *Staphylococcus aureus* [1] and, occasionally, coagulase-negative staphylococci (CNS) carrying the enterotoxin genes [2]. SFP is usually acute, and symptoms, including diarrhea, vomiting, abdominal pain, and nausea, can be observed approximately 1–2 h after ingestion [3]. SFP may not be lethal, but elderly individuals are more sensitive to toxic substances than younger people are; therefore, SFP can be the cause of death in elderly patients with gastroduodenitis [4].

SFP can occur in any type of food. The pathogen, which is usually transferred to food from food handlers, produces SEs if the conditions are suitable for bacterial growth [5]. A previous study in the UK reported that the most common contaminated foods were meat products (75%), followed by fish and shellfish (7%) and dairy products (8%) [6].

The presence of staphylococci in raw milk generally comes from cows with mastitis, food handlers, or deficient hygiene [7]. Although pasteurization can destroy staphylococci, some SEs are still biologically active as they are resistant to pasteurization temperatures [8] and even temperatures higher than 100 °C [9]. Therefore, even if heat treatment eliminates or reduces staphylococci to minimal bacterial counts in milk, contaminated milk can still cause SFP because of the presence of heat-stable SEs. This emphasizes the importance of storing raw milk in appropriate storage conditions to prevent enterotoxin production. Normally, enterotoxin production occurs between 10 and 46 °C [10]. Higher temperatures during prolonged storage can cause an increase in the amount of toxin produced by the contaminating bacteria. Thus, because of the importance of these toxins in public health, an efficient screening method to detect the prevalence of toxic strains of staphylococci in bovine milk is required.

Currently, more than 20 SEs have been identified, which can be divided into two types: (1) classical SEs consisting of *sea, seb, sec, sed*, and *see*; (2) new enterotoxins consisting of SEG–SElZ [11]. Classical SEs are important and are a major cause of SFP, accounting for over 90% of SFP cases worldwide [3,12]. SEA is the most common cause of food poisoning in the United States, accounting for up to 77.8% of all SFP cases, followed by SED (37.5%) and SEB (10%). In addition, *sea, seb, sec, sed*, and *see* genes exhibit different patterns of expression of SEs. The *sea* gene is usually expressed in the mid-exponential phase of growth, whereas the expression of *seb, sec*, and *sed* genes is regulated by an accessory gene regulator (*agr*) [13]. Therefore, it is necessary to study the expression of individual SE genes during growth, especially in milk, which is a common source of staphylococcal contamination.

The aims of this study were: (1) to investigate classical SE genes, *sea, seb, sec, sed*, and *see*, in *S. aureus* and CNS isolated from milk samples obtained from bovine with mastitis; (2) to determine the effect of temperature on the expression of classical SE genes of staphylococci inoculated in milk.

## 2. Results

### 2.1. Distribution of Classical SE Genes among Staphylococcal Isolates

Among the 98 staphylococcal isolates, 51 were confirmed as *S. aureus*, whereas 47 were confirmed as CNS. The most common species among CNS isolates was *S. chromogenes*, followed by *S. haemolyticus* and *S. epidermidis*, as shown in Table 1.

The classical SE genes among *Staphylococcus* species were confirmed using monoplex PCR. Among all isolates, 24.49% of isolates (24/98) harbored at least one SE gene, with *sec* being the most commonly detected SE gene (21/98, 21.43%). The *sed* gene was not detected in any of the staphylococcal isolates included in this study. Among the *S. aureus* isolates, 35.30% (18/51) harbored at least one SE gene. The most frequent gene profile was *sec* (29.41%, 15/51), followed by *sea + sec* (5.88%, 3/51) (Table 1). Classical SE genes were detected in 12.77% (6/47) of CNS isolates, with *sec* being the most prevalent (6.38%, 3/47), followed by *see* (4.26%, 2/47) and *seb* (2.13%, 1/47) (Table 1).

### 2.2. Growth of Staphylococci in Milk

Four staphylococcal isolates carrying *sea, seb, sec*, and *see* genes were inoculated into milk subjected to ultra-high-temperature (UHT) processing and incubated at two different temperatures to determine the growth curves. In milk inoculated with the *sea*-positive, *sec*-positive, and *see*-positive strains, the concentrations of inoculated strains incubated at room temperature increased with time and were significantly higher than those of strains incubated at 8 °C for 4 to 48 h of incubation (*p* < 0.01). In milk inoculated with the *seb*-positive strain, the concentrations of the inoculated strain incubated at room temperature were significantly higher than those of strains incubated at 8 °C for 8 to 48 h (*p* < 0.05). The growth curves of *sea*-positive, *seb*-positive, *sec*-positive, and *see*-positive staphylococci incubated at 8 °C and room temperature are shown in Supplementary Figure S1.

### 2.3. Expression of Classical SE Genes

**Figure 1:**
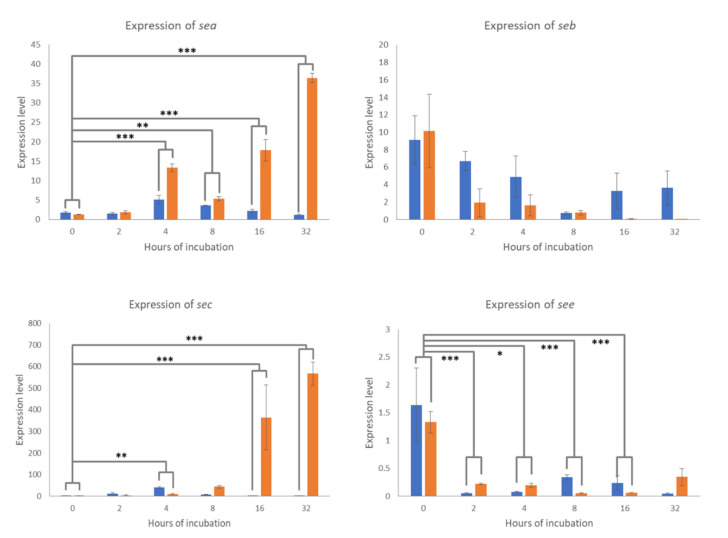
Relative expression levels of *sea, seb, sec,* and *see* genes of staphylococcus in milk. Asterisks indicate statistically significant comparisons of the differences of the relative expression levels between 8 °C (blue bar) and room temperature (orange bar) at 0 h of incubation, and the differences of the relative expression levels between 8 °C and room temperature at 2, 4, 8, 16, and 32 h of incubation. Significant codes: * indicates *p* < 0.05, ** indicates *p* < 0.01, *** indicates *p* < 0.001.

The relative expression (RQ) levels of the *sea, seb, sec*, and *see* genes of staphylococci inoculated into the UHT milk incubated at 8 °C and room temperature for 0, 2, 4, 8, 16, and 32 h of incubation were measured, calculated, and compared. The expression levels of *sea* and *sec* genes of staphylococci incubated at 8 °C were very low throughout the 32-h incubation period. In contrast, the expression levels of these two genes at room temperature gradually increased as the incubation time increased and were higher than those at 8 °C as shown in Figure 1. As the post hoc pairwise comparison of the temperature–incubation time interaction, the difference between the relative expression at 8 °C and room temperature at 0 h of incubation was compared with those differences at 2, 4, 8, 16, and 32 h of incubation. The difference between the relative expression of the *sea* gene at 8 °C and room temperature at 0 h of incubation was significantly lower than those differences at 4, 8, 16, and 32 h of incubation (*p* < 0.05), as shown in Figure 1. Similarly, the difference between the relative expression of the *sec* gene at 8 °C and room temperature at 0 h of incubation was significantly lower than those differences at 4, 16, and 32 h of incubation (*p* < 0.05), as shown in Figure 1. In contrast to the expression levels of *sea* and *sec* genes, the expression levels of *seb* and *see* genes in milk incubated at both temperatures were low and gradually decreased throughout the 32-h incubation period, as shown in Figure 1. No significant comparison was observed when the difference between the relative expression of the *seb* gene at 8 °C and room temperature at 0 h of incubation was compared to those differences at 4, 8, 16, and 32 h of incubation (*p* > 0.05), as shown in Figure 1. However, the difference between the relative expression of the *see* gene at 8 °C and room temperature at 0 h of incubation was significantly different from those differences at 2, 4, 8, and 16 h of incubation (*p* < 0.05), as shown in Figure 1.

## 3. Discussion

*S. aureus* and CNS are common pathogens that cause bovine mastitis in different regions worldwide [14,15]. Infected udders can release large amounts of these bacteria into the milk, which can increase the risk of SFP, as the bacteria carrying the SE genes may produce the SEs into the milk. The ability of *S. aureus* and CNS to produce SEs in milk depends on whether or not the strain is carrying the SE genes and whether the environmental conditions are suitable for SE synthesis.

The aim of this study was to determine the effect of temperature (cool and ambient) on the expression of classical SE genes in staphylococci in milk. The growth of staphylococcal isolates inoculated in UHT milk was determined. Inoculated milk samples were incubated at 8 °C and room temperature to simulate the appropriate and inappropriate storage conditions, respectively. The sterility of the UHT milk and the microbiological media was critical for the growth curve interpretation. In the current study, the UHT milk used in the experiment was sterile without any observed bacterial growth before inoculation. In contrast, the sterility control for the microbiological media as an indicator of the environmental control was not performed. However, during the experiment of growth curve evaluation of staphylococci in milk, homogeneous colonies of staphylococci were observed from all milk drops of all dilutions. No signs of contamination were observed throughout the experiment.

The results of this study showed that more than one-third (35.30%) of *S. aureus* isolates were positive for classical SE genes. Similar findings were also reported in Brazil (35%) [16] and Poland (32%) [17]. In contrast, only 12.7% of the CNS were reported to carry classical SE genes in this study. Several studies also reported low detection rates of SE genes among CNS, which ranged from 5.3 to 19.4% [17,18,19]. These findings indicate that the contamination of milk by *S. aureus* can pose a higher risk of SE contamination and SFP in consumers compared to that by CNS. Therefore, in regions where the prevalence of *S. aureus* contamination in milk is high, more attention should be paid towards the raw milk handling process to reduce the risk of SFP.

Among the CNS species identified in the current study, only three species, including *S. chromogenes, S. epidermidis*, and *S. haemolyticus*, were found to carry SE genes. Different species of CNS have previously been reported to carry SE genes, such as *S. cohnii, S. epidermidis*, and *S. haemolyticus* in Spain [20]; *S. chromogenes*, *S. warneri, S. sciuri*, and *S. saprophyticus* in Germany [21]; and *S. epidermidis* and *S. warneri* in Brazil [16].

The most frequent SE genes detected in this study were *sec* followed by *sea*. This result is similar to that of several previous studies, where the *sec* gene has been reported as the most common classical SE gene detected among *S. aureus* isolates causing bovine mastitis [22,23,24]. In addition, Piechota et al. [17] reported that *sec* was the most prevalent SE gene among CNS. Various studies in different regions have also revealed that *sea* was the most common bovine mastitis-associated SE gene among *S. aureus* [25,26] and CNS [4,21]. A recent study revealed that *sec* is an important virulence factor of *S. aureus* that causes inflammation and tissue damage to the infected mammary gland [27]. Therefore, *sec* may play a critical role in bovine mastitis, which may explain the high detection rate of the *sec* gene among the pathogenic staphylococci causing bovine mastitis in the current study.

The current study demonstrated that the growth of staphylococci in milk incubated at room temperature was faster than that at 8 °C, especially when the milk was incubated for a long period of time (4–48 h). In agreement with the growth of staphylococci in milk, the expression levels of *sea* in the inoculated milk incubated at room temperature increased with time and were higher than those in milk incubated at 8 °C. A similar finding was reported by Babić et al. (2019), wherein the growth rate, *sea* gene expression, and SEA production were affected by the type of milk, the storage time, and the storage temperature [28]. The *sea* gene was found to be located in a family of temperate bacteriophages [29]. The production of SEA was reported to be associated with the phage’s life cycle [30] and the bacterial stress [31]. The SEA production is increased during the exponential growth phase and highest at the peak of replication [32]. In general, SEA production could be detected at 10 to 45 °C, which was increased as the temperature increased [33]. Therefore, to minimize the risk of SEA contamination in milk, good hygiene practices to avoid staphylococcal contamination, as well as maintaining the cold temperature throughout the production chain of dairy products, are critical.

In the current study, an extremely high expression of the *sec* gene was observed in milk inoculated with *S. aureus* at 16 and 32 h of incubation at room temperature. This upregulation of *sec* gene did not linearly correlate with the growth rate of the inoculated *sec*-positive strain under the same conditions. This finding did not agree with the findings by [34], which demonstrated the decreased expression of the *sec* gene in milk after 20 h of incubation at room temperature [34]. In contrast to the expression of *sec*, Valihrach et al. [34] reported an increased *sec* production in milk after 20 h of incubation at room temperature, although it was lower than those in cultured broth. Likewise, another study also reported the increased production of SEC_bovine_ in milk after 18 h of incubation at 25 °C [35]. The expression of the *sec* gene is reported to be regulated by the accessory gene regulator (Agr) quorum sensing (QS) system, which is activated when the cell density is high. Until now, up to seven subtypes of SECs have been discovered [36,37]. The genes encoding these SEC variants are very similar and can be differentiated only by *sec* gene sequencing. Moreover, the variation of growth and SEC production among different strains of *S. aureus* inoculated in milk has been reported [38]. Therefore, the contrasting findings on the expression of the sec gene in milk reported in the current study and the study by [34] could be due to the variation of *S. aureus* strains carrying the *sec* gene specific for different SEC subtypes. The sequence of *sec* gene and the production of SEC should be further investigated in future studies. In addition, the *sec* gene is located on the mobile genetic element called “pathogenicity islands” on chromosomes [39]. Some strains may contain more than one pathogenicity island, and some pathogenicity islands can have one or more virulence genes [40]. Therefore, we propose that the extremely high expression level of *sec* observed in this study may be due to the high copy numbers of the *sec* gene in the chromosome of the inoculated staphylococcal strain. However, this hypothesis needs to be validated in future studies.

In the current study, the expression levels of *seb* and *see* in milk were much lower than those of *sea* and *sec*. Similar to the *sec* gene, the *seb* gene could be detected on a pathogenicity island or a plasmid and was regulated by the Agr QS system [41]. On the other hand, the *see* gene was identified on a defective prophage [42]. Like SEA, SEE contains 90% amino acid identical to SEA [43] and was not associated with the bacterial growth [44]. According to the current study, even though the exponential growth of staphylococcal strains carrying *seb* and *see* were observed in milk, the expression of these two genes seemed to be inhibited in milk. The detected higher levels of *seb* and *see* gene expression immediately after milk inoculation could be from the trace of the gene expression in the inoculated cultured media. The findings in the current study indicated that some factors in milk can regulate the expression of the *seb* and *see* genes. The behavior of these two genes and the production of SEB and SEE in milk should be further investigated.

*S. aureus* and CNS are common causative agents of mastitis in dairy herds. The findings in the current study indicated that the presences of bovine mastitis caused by these bacteria do not only impair animal health, but they also increased the risks of human health. Therefore, the control of SFP could be relied on the One Health approach, which refers to the collaborative problem solving used to ensure the well-being of people, animals, and the environment [45]. Farmer and veterinarian collaborations to control bovine mastitis in dairy herds should be emphasized to reduce the contamination of the microbial agents at the farms. Good hygiene on the farms and appropriate milk handlings and storage along the production line of dairy products should not be overlooked and should be regularly monitored to ensure the safety of the consumer’s health.

## 4. Materials and Methods

### 4.1. Bacterial Isolates

Staphylococcal isolates were obtained from the archived collection of the central laboratory, Faculty of Veterinary Medicine, Chiang Mai University, Chiang Mai, Thailand, and they were stored at −80 °C. Only staphylococcal isolates primarily cultured from quarter milk samples of clinical and subclinical bovine mastitis cases in Chiang Mai province, Thailand, as part of some previous projects during 2013 to 2017 (*n* = 98) were included in the study.

### 4.2. Characterization of the Genus Staphylococcus

All 98 frozen isolates were regrown on 5% bovine blood agar and incubated at 37 °C for 24 h. Colonies on blood agar were observed for their morphologies and hemolysis. All isolates were presumptively confirmed as members of the genus Staphylococcus using Gram staining and a catalase test. Gram-positive cocci bacteria with catalase positive reactions were presumptively characterized as staphylococci. A coagulase test was used to characterize coagulase-positive and coagulase-negative staphylococci as suggested by the National Mastitis Council [46]. Then, all isolates were subjected for genotyping identification using amplification of the *nuc* gene, for *S. aureus* identification, and the 16S rRNA gene sequencing technique, for species identification of CNS.

### 4.3. Genomic DNA Extraction

All 98 staphylococcal isolates were inoculated in 5 mL of brain heart infusion broth (BHI, Wahana Hilab, Yogyakarta, Indonesia) and incubated at 37 °C for 24 h. One milliliter of the overnight culture in BHI broth was centrifuged at 10,000× *g* for 5 min. The supernatant was removed, and the pellet was washed with DEPC-treated water. The tube was then centrifuged at 10,000× *g* for 5 min. After centrifugation, the supernatant was removed, and the cell pellet was used for DNA extraction. Genomic DNA of staphylococci was extracted using a DNA extraction kit (NucleoSpin™, Macherey-Nagel GmbH & Co.KG, Dueren, Germany) according to the manufacturer’s instructions and stored at −20 °C until use.

### 4.4. Genotypic Identification of Staphylococci

Amplification of the *nuc* gene was adopted to identify *S. aureus* isolates. The *nuc* gene was amplified by monoplex polymerase chain reaction (PCR) with all 98 staphylococcal isolates. The PCR mixture consisted of 1 μL of DNA template, 12.5 μL of 2X MyTaq™ Red Mix (Meridian Bioscience, London, UK), 20 μM of each primer, and sterile water to a total volume of 25 μL. The PCR cycle started with an initial denaturation step (95 °C, 5 min), followed by 34 cycles of denaturation at 95 °C for 1 min, annealing at the appropriate annealing temperature (Supplementary Table S1) for 50 s, and extension at 72 °C for 5 min, followed by a single final extension at 72 °C for 5 min. In each PCR run, a negative control consisting of the same PCR mixture without DNA was used. All PCR products were electrophoresed using a 1.5% agarose gel containing 1 μg/mL ethidium bromide. The information on all primers and their references are provided in Supplementary Table S1. *S. aureus* DMST 4745 (ATCC 29123) and *S. epidermidis* DMST 15505 (ATCC 12228) were used as positive and negative control strains for *nuc* gene amplification, respectively. Only coagulase-positive staphylococci containing *nuc* gene would be identified as *S. aureus* [47]. All *nuc*-negative samples were selected for speciation using 16S rRNA gene sequencing [47]. Monoplex PCR amplification of the 16S rRNA gene was performed using all *nuc*-negative samples. For speciation, PCR products of the 16S rRNA gene were sequenced. The sequences were compared with sequences from the GenBank database using BLAST [48].

### 4.5. Detection of Classical SE Genes

Five monoplex PCRs amplifying five classical SE genes, including *sea, seb, sec, sed*, and *see* (Supplementary Table S1), were performed with each isolate. Similar PCR mixture and conditions, as described in the previous section, were adopted for the detection of classical SE genes.

### 4.6. Growth Curve Evaluation of Staphylococci in Milk

An isolate carrying each of the detected classical SE genes was selected and regrown in 10 mL of BHI broth. After 8 h of incubation, 5 mL of the incubated broth was centrifuged at 10,000× *g* for 5 min. The supernatant was removed, and the pellet was washed twice with 1 mL of 0.85% NaCl solution. The concentration of bacteria was adjusted to 0.3 OD_600nm_ (2–9 × 10^8^ CFU/mL) using 0.85% NaCl. Subsequently, the bacterial solution (2.5 mL) was inoculated into 247.5 mL of UHT milk. The inoculated milk was divided into two flasks containing 125 mL each. One flask was stored at 8 °C, whereas the other flask was stored at room temperature (approximately 25 °C). Bacterial growth in milk was monitored at 0, 2, 4, 8, 16, 32, and 48 h of incubation at both incubation temperatures. The concentration of bacteria (CFU/mL) in each sample at each time point and each incubation temperature was determined using the drop plate method [49]. Briefly, 1 mL of the inoculated milk was transferred to 9 mL of 0.85% NaCl. Ten-fold dilutions were prepared from 10^−1^ to 10^−8^ in 0.85% NaCl. Then, 10 µL of each dilution (3 drops) were dropped onto the nutrient agar. The agar plates were incubated at 37 °C for 15 h before enumeration. For sterility control, the UHT milk at 0 h of incubation was cultured, and the bacterial growth in the UHT milk must not be presented prior to interpreting the growth curve. Moreover, the observed bacterial colonies during the growth curve experiment must be pure (homogeneous colonies) without any signs of contamination to ensure the purity of inoculated strain throughout the 48 h of incubation.

### 4.7. RNA Extraction and cDNA Synthesis

Total RNA was extracted from inoculated milk incubated at 8 °C and room temperature at 0, 2, 4, 8, 16, and 32 h of incubation. One milliliter of inoculated milk was centrifuged at 10,000× *g* for 5 min to separate the milk fat. After removing the milk fat and the supernatant, the pellet was suspended in 180 µL of cell lysis buffer (20 mM Tris/HCl, 2 mM EDTA, 1% Triton X-100 (pH 8), supplemented with 40 mg/mL lysozyme). Total RNA was extracted using the RiboZol™ RNA extraction reagent (VWR Life Science, Solon, OH, USA) according to the manufacturer’s protocol. The extracted total RNA was eluted with 50 μL of DEPC water. The total RNA concentration was determined using a NanoDrop spectrophotometer at OD_260/280 nm_ (Thermo Fisher Scientific, Waltham, MA, USA). Reverse transcription was performed to generate complementary DNA (cDNA) using the Tetro^™^ cDNA synthesis Kit (Meridian Bioscience, London, UK) according to the manufacturer’s protocol. Total cDNA was synthesized using 2 μg of total RNA in 20 μL of the reverse transcription reaction mixture. The cDNA was then stored at −20 °C until use.

### 4.8. Quantitative Real-Time PCR (qPCR) Assay

qPCR analysis was performed using an Applied Biosystem 7300 instrument (Thermo Fisher Scientific). The primers for the detection of 16S rRNA (housekeeping gene) and classical SE genes (*sea, seb, sec,* and *see*) are shown in Supplementary Table S2. The qPCR reaction mixture consisted of 10 μL of SYBR Green PCR Master Mix (SensiFAST^TM^, Meridian Bioscience, London, UK), 1 μL of cDNA, 10 μmol of each forward and reverse primers, and nuclease-free water to a total volume of 20 μL. The samples were placed in triplicate on 96-well plates and covered with an optical adhesive. The qPCR program was as follows: 50 °C for 2 min, initial denaturation at 95 °C for 2 min, followed by 40 cycles of denaturation at 95 °C for 8 s and annealing/extension at 60 °C for 30 s. A melting curve was used to check for non-specific products. The relative expression of the classical SE genes was calculated based on the relative quantification (RQ), where the 16S rRNA gene was used as an endogenous control for each enterotoxin gene. All experiments were performed three times.

### 4.9. Statistical Analyses

The detection rate of each classical SE gene among *S. aureus* and CNS was descriptively reported as a percentage. Staphylococcal counts (CFU/mL) were transformed to log_10_ CFU/mL. Mixed model analysis of variance (ANOVA) was used to determine the effect of temperature and incubation time on the concentration of bacteria (log_10_ CFU/mL) of each isolate. Mixed model ANOVA was performed using the “lmerTest” package [50]. The Tukey method of pairwise comparison was performed using the “lsmeans” package [51]. The Aligned Rank Transform (ART) for nonparametric factorial data analysis was used to determine the effect of temperature, incubation time, and the temperature–incubation time interaction on the expression levels (RQ) of each classical SE gene. The ART for nonparametric factorial data analysis and the post hoc pairwise comparisons of significant main effects were performed using the “ARTool” package [52,53]. The post hoc pairwise comparisons of significant interactions were performed using the “phia” package [54]. When the temperature–incubation time interaction was significant, the difference between the relative expression at 8 °C and room temperature at 0 h of incubation was compared with those differences at 2, 4, 8, 16, and 32 h of incubation. Statistical significance was set at *p* < 0.05. All statistical analyses were performed using the R statistical software [54].

## 5. Conclusions

The results of this study revealed that the classical SE genes were more prevalent among *S. aureus* than among CNS isolated from bovine milk. The most common enterotoxin coding genes were *sec* followed by *sea, see*, and *seb*. After 16–32 h of incubation, the staphylococci in milk stored at room temperature showed significantly higher growth rates and higher *sea* and *sec* gene expression than those stored at 8 °C. The results of this study emphasize the importance of storing milk at temperatures below 8 °C to reduce the risk of SFP.

## Figures and Tables

**Table 1:** Distribution of classical staphylococcal enterotoxin genes among staphylococci associated with bovine mastitis in northern Thailand.

Identification (*n*)	Number of Isolates without Detected Gene	Number of Isolates Carrying Gene					
		*sea*	*seb*	*sec*	*sed*	*see*	*sea + sec*
*S. aureus* (51)	33	–	–	15	–	–	3
*S. chomogenes* (24)	21	–	–	3	–	–	–
*S. haemolyticus* (7)	6	–	–	–	–	1	–
*S. epidermidis* (5)	3	–	1	–	–	1	–
Other CNS (11)	11	–	–	–	–	–	–
Total (98)	74	–	1	18	–	2	3

## Data Availability

The data presented in this study are available on request from the corresponding author.

## Supplementary Materials

The following are available online at https://www.mdpi.com/article/10.3390/pathogens10080975/s1, Figure S1: Growth curve of sea-, seb-, sec-, and see positive staphylococci in UHT milk incubated at 8 °C and room temperature, Table S1: Sequences of PCR primers used to identify Staphylococcus species and detect classical staphylococcal enterotoxin genes, Table S2: Sequences of qPCR primers used to estimate the expression of classical staphylococcal enterotoxin genes.
