# Supplementary material for: Effect of Temperature on the Expression of Classical Enterotoxin Genes among Staphylococci Associated with Bovine Mastitis
Source: Pathogens. 2021 Aug 2;10(8):975. doi: 10.3390/pathogens10080975 (PMC8398761; doi:10.3390/pathogens10080975)
Supplement: Supplementary file 1 [file pathogens-10-00975-s001.zip › pathogens-1284101-supplementary.pdf]

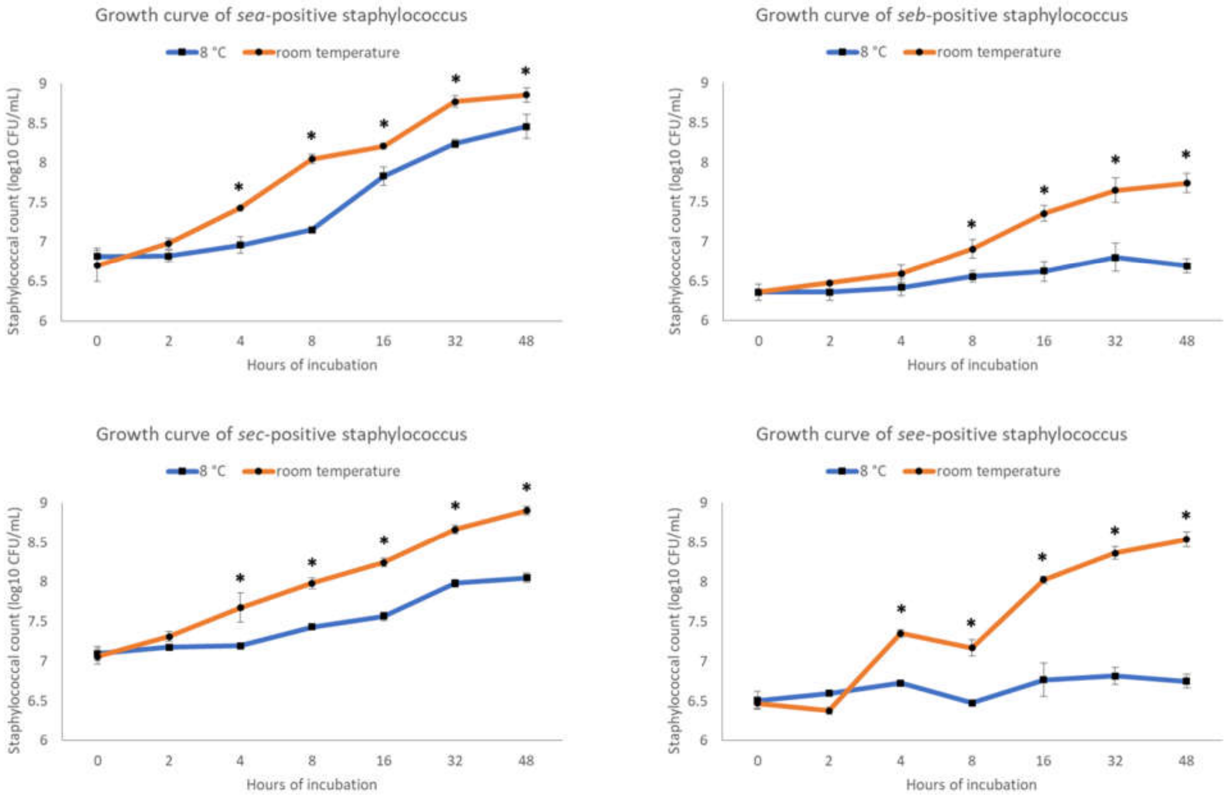

Figure S1. Growth curve of *sea-*, *seb-*, *sec-*, and *see* positive staphylococci in UHT milk incubated at 8 °C and room temperature. Asterisks indicate statistically significant comparisons of staphylococcal count at both temperature at each hour of incubation.

Table S1. Sequences of PCR primers used to identify *Staphylococcus* species and detect classical staphylococcal enterotoxin genes.

**Gene**	**Primer**	**Oligonucletides sequence (5′-3′)**	**Product (bp)**	**Annealing temperature**	**References**
sea	sea-F	CAGCATACTATATTGTTTAAAGGC	400	53 °C	[1]
	sea-R	CCTCTGAACCTTCCCATC			
seb	seb-F	TCGCATCAAACTGACAAACG	478	50 °C	[2]
	seb-R	GCAGGTACTCTATAAGTGCC			
sec	sec-F	CTCAAGAACTAGACATAAAAGCTAGG	271	53 °C	[3]
	sec-R	TCAAAATCGGATTAACATTATCC			
sed	sed-F	CTAGTTTGGTAATATCTCCTTTAAACG	319	53 °C	[3]
	sed-R	TTAATGCTATATCTTATAGGGTAAACA TC			
see	see-F	CAGTACCTATAGATAAAGTTAAAACAAG	178	53 °C	[3]
	see-R	CTAACTTACCGTGGACCCTTC			
nuc	nuc-F	GCGATTGATGGTGATACGGTT	279	62 °C	[4]
	nuc-R	AGCCAAGCCTTGACGAACTAAAGC			
16S rRNA	16S-F	AACTCTGTTATTAGGGAAGAAGAACA	756	62 °C	[4]
	16S-R	CCACCTTCCTCCGGTTTGTCACC			

Table S2. Sequences of qPCR primers used to estimate the expression of classical staphylococcal enterotoxin genes.

Gene	Primer	Oligonucletides sequence (5′-3′)	Product (bp)	References
sea	sea-F	AAAATACAGTACCTTTGGAAACGGTT	92	[5]
	sea-R	TTTCCTGTAAATAACGTCTTGCTTGA		
seb	seb-F	ACACCCAACGTT TTAGCAGAGAG	81	[5]
	seb-R	CCATCAAACCAGTGAATTTAGTCG		
sec	sec-F	AATAAAACGGTTGATTCTAAAAGTGTGAA	80	[5]
	sec-R	ATCAAAATCGGATTAACATTATCCATTC		
see	see-F	TCAATGTGCTGGAGGCACACCAA	51	[6]
	see-R	ACACCCCCGTACATA CATGCTGTT		
16S rRNA	16S-F	AAGTCCCGCAACGAGCGCAA	86	[6]
	16S-R	CCTCCGGTTTGTCACCGGCA		

References

1. Park, J.Y.; Fox, L.K.; Seo, K.S.; McGuire, M.A.; Park, Y.H.; Rurangirwa, F.R.; Sischo, W.M.; Bohach, G.A. Detection of Classical and Newly Described Staphylococcal Superantigen Genes in Coagulase-Negative Staphylococci Isolated from Bovine Intramammary Infections. *Vet. Microbiol.* **2011**, *147*, 149–154.
2. Johnson, W.M.; Tyler, S.D.; Ewan, E.P.; Ashton, F.E.; Pollard, D.R.; Rozee, K.R. Detection of genes for enterotoxins, exfoliative toxins, and toxic shock syndrome toxin 1 in *Staphylococcus aureus* by the polymerase chain reaction. *J. Clin. Microbiol*. **1991**, *29*, 426–430.
3. Becker, K.; Roth, R.; Peters, G. Rapid and specific detection of toxigenic *Staphylococcus aureus*: use of two multiplex PCR enzyme immunoassays for amplification and hybridization of staphylococcal enterotoxin genes, exfoliative toxin genes, and toxic shock syndrome toxin 1 gene. *J. Clin. Microbiol.* **1998**, *36*, 2548–2553.
4. Ciftci, A.; Findik, A.; Onuk, E.E., Savasan, S. Detection of methicillin resistance and slime factor production of *Staphylococcus aureus* in bovine mastitis" *Braz. J. Microbiol*. **1958**, *40*, 254–261.
5. Klotz, M.; Opper, S.; Heeg, K.; Zimmermann, S. Detection of *Staphylococcus aureus* enterotoxins A to D by real-time fluorescence PCR assay. *J. Clin. Microbiol*. **2003**, *41*, 4683–4687.
6. Bastos, C.P.; Bassani, M.T.; Mata, M.M.; Lopes, G.V.; da Silva, W.P. Prevalence and expression of staphylococcal enterotoxin genes in Staphylococcus aureus isolated from food poisoning outbreaks. *Can. J. Microbiol*. **2017**, *63*, 834–840.
